# The Challenge of Subjective Cognitive Complaints and Executive Functions in Middle-Aged Adults as a Preclinical Stage of Dementia: A Systematic Review

**DOI:** 10.3390/geriatrics7020030

**Published:** 2022-03-08

**Authors:** Felipe Webster-Cordero, Lydia Giménez-Llort

**Affiliations:** 1 Department of Psychiatry and Forensic Medicine, School of Medicine, Universitat Autònoma de Barcelona, E-08193 Barcelona, Spain; 2 Hospital Santa Inés, Cuenca 010107, Ecuador; 3 Institut de Neurociències, Universitat Autònoma de Barcelona, E-08193 Barcelona, Spain

**Keywords:** subjective cognitive complaints, stereotypes, aging, complaints, executive functions, memory, mild cognitive impairment, dementia

## Abstract

Subjective cognitive complaints correspond to a heterogeneous construct that frequently occurs in the early stages of older adult life. Despite being a common source of worry for middle-aged people, it can be underestimated when clinical and neuropsychological assessments discard any underlying pathological processes. Negative age stereotyping but also self-stereotyping can contribute to doing so. Although its diagnosis is a challenge, its implication as a possible predictor of mild cognitive impairment or dementia increases the interest in its early diagnosis and intervention. The present systematic review analyzes the empirical data on the relationship between these complaints and early executive dysfunction with possible predictive value for preclinical stages of dementia. The sixteen papers obtained from the PubMed and Embase databases were exploratory, cross-sectional and prospective in scope. The studies corroborated the relationship between subjective cognitive complaints and some executive processes, which is noteworthy since many people with subjective executive complaints progress to dementia. The relational studies confirmed that impaired executive performance is associated with CSF biomarkers and reduced cortical volume in specific brain regions. However, the heterogeneity of reports in these studies demands stronger efforts in future research with specific tools applied in clinical and neuropsychological assessments and analyzed under a gender perspective.

## 1. Introduction

With the aging population, geriatricians highlight the relevance of disease prevention and diagnosis of old-age-specific diseases and the best use of healthcare professionals’ specialties [1]. However, in this context, age stereotypes are known to influence behavioral, physical and cognitive outcomes among healthy older adults [2,3,4]. Moreover, the health care professionals’ beliefs and attitudes toward older people may also exert a significant impact [5]. Thus, stigmatization of older people has been shown to induce the overestimation of their age-related cognitive decline and the common perception that older people have worse cognitive competencies [2]. Growing evidence shows that these negative aging stereotypes impair the performance of healthy older adults on cognitive tests, while positive age stereotyping exerts less intense effects [2]. Thus, the impact of the age-based stereotype threat (the threat of being judged stereotypically) on older adults’ episodic and working memory, false memory susceptibility and subjective age has been recently reported [6,7,8,9,10].

Research on the role of age stereotypes and subjective aging in health across the life span identifies several theoretical approaches for potential mechanisms to explain how personal views of aging might influence health-related outcomes in later life [11]. In fact, self-stereotyping also has long-term negative effects. According to the internalization hypothesis, holding more negative age stereotypes and perceiving more age discrimination are associated with feeling older, lower self-esteem and worse perceptions of one’s own aging [7]. Similarly, internalized negative age stereotypes and subjective cognitive impairment have been related to increased depressive effects despite not being related to age-related dysregulations of cortisol [12]. In contrast, essentialist beliefs about aging seem to moderate the impact of negative age stereotypes on older adults’ performance and physiological reactivity [13].

Despite most research on age stereotypes being focused on the above-mentioned external/internal distortions referred to the older adult, nihilist aptitudes towards the inherent limitations of advanced aging and underestimations during middle adulthood can also occur [5]. Gerontologists warn that negative aging stereotypes can decrease performance on short cognitive tests widely used in primary care to screen for predementia [14]. However, on the other hand, the cognitive decline that occurs after the age of 50 or 60 is accompanied by minimal changes in some cognitive processes that are, in many cases, self-perceived by the individual but yet undetectable by classical neuropsychological assessments primarily focused on declarative memory. Hence, the term subjective cognitive impairment (SCI) associated with a subjective decline in one or more cognitive areas (attention, memory, reasoning, language, etc.) is not objectively measurable after a neurocognitive assessment despite being reported by the subject or a close person [15]. Its possible relationship with dementia points to this challenging SCI diagnosis and understanding as a subject of growing research interest, since it could have a crucial role in applying early and personalized preventive and therapeutic interventions [16,17].

An increasing number of young adults calls at clinical centers for clinical consultation due to problems in their cognitive performance, reporting problems with memory, concentration, or reasoning. In the case of middle-aged adults, this awareness usually becomes a source of worry about a predictive value for a worse clinical condition, a prelude to dementia. People’s subjective perception of their cognitive performance is highly variable and the heterogeneity of these subjective cognitive complaints makes differential diagnosis a challenge [18,19].

In fact, cognitive symptomatology in adults may be directly related to depressive symptoms or personality characteristics that influence their perception of their cognitive performance [20,21]. Moreover, the sum of these factors may be a predictor of a negative perception of quality of life [22]. Therefore, in many cases, neuropsychological assessment discards the existence of an actual cognitive impairment underlying the subjective complaints [23]. In the current pandemic scenario, the number of middle-aged adults with cognitive complaints has increased dramatically, becoming a topic of growing interest [24,25]. This subclinical condition has been usually exclusively associated with loss of memory processes, perhaps due to the lack of specificity when assessing neurocognitive processes. In this sense, questionnaires or standardized tests to assess subjective memory complaints are not analyzed in detail; many of them do not assess all the cognitive processes involved or do not assess qualitative aspects, via which clinically significant data are obtained [26,27].

Due to the significant heterogeneity of the neurocognitive processes involved in subjective cognitive impairment, the neuropsychological assessment is an essential diagnostic tool together with the multi- and interdisciplinary clinical study [28]. There are conflicting data on how this symptomatology manifests and whether it can be determined as a syndrome distinct from mild cognitive impairment (MCI) and dementia [29]. However, although there is not a unified criterion for this entity yet, biomarkers and evidence of anato-functional changes through neuroimaging studies are now known [30,31]. Regarding the dysfunctional neuroanatomical substrate, frontal executive processes seem to be, in part, the target of several lines of research where they are analyzed as possible initial symptoms of this not yet determined complex clinical picture. Therefore, the present review aims to systematically analyze the emerging empirical data on the relationship between subjective cognitive complaints (SCCs) and executive functions in middle-aged adults to help understand possible deficits at this level in the preclinical stages of dementia. This would also provide evidence to counteract the underestimation of SCCs before clinical diagnosis using the biomedical model.

## 2. Materials and Methods

**Figure 1:**
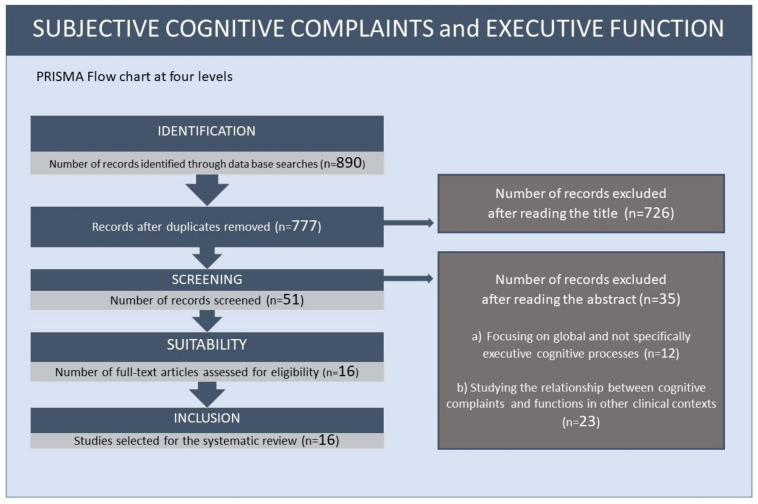
Flow chart of the systematic review on subjective cognitive complaints and executive functions.

In the present study, a systematic review on the relationship between subjective cognitive complaints and executive functions was carried out, following the guidelines of the PRISMA [32] method for its correct elaboration (see Figure 1).

### 2.1. Systematic Search (Databases, Descriptors, Search Formulas)

Through the systematic search, scientific publications from 2005 (when the literature on “subjective complaints” started to grow) to the present on the chosen topic were analyzed, using the PubMed and Embase databases as the primary tool. The terms “subjective”, “memory”, “cognitive”, “complaints”, “executive functions” and “working memory” used in the search with the Booleans “AND” and “OR” yielded 890 results as possible sources of analysis, after which inclusion and exclusion criteria were established.

### 2.2. Inclusion Criteria

1. Empirical research on the relationship between subjective cognitive complaints and executive functions in preclinical stages of dementia;
2. Papers published from 2005 to the present and with a study population specificity of 60 years of age and older.

### 2.3. Exclusion Criteria

3. Papers addressing the topic of subjective cognitive complaints in a general or specific way in other clinical contexts;
4. Studies analyzing executive functions in stages where there is already a diagnosis of dementia in advanced stages.

### 2.4. Flow Chart

Following these criteria, on reading the title of the initially selected articles, 51 papers were chosen. After reading the abstract, 35 were discarded; some focused on global cognitive processes and not specifically executive ones (*n* = 12) and others studied the relationship between subjective cognitive complaints and executive functions in other clinical contexts (*n* = 23). Finally, 16 articles were selected for review, as they focused on studying the relationship between some executive processes with SCCs and their involvement in the subclinical stages of mild cognitive impairment and dementia.

## 3. Results

Table 1 summarizes the empirical studies and depicts the main findings on the relationship between subjective cognitive complaints and executive functions.

The number of studies on the relationship between subjective cognitive complaints and executive functions was scarce, but the overall data are based on a total sample of 5137 participants gathered from western (USA, Australia, and Central and Southern Europe) and oriental (Korea) countries. The sociodemographic variables included sex (with a 1:3 Women:Men ratio) and years of education, but none analyzed the data on subjective complaints in a segregated manner. All the studies confirmed the hypothesis of a direct link between these two psychological constructs, providing evidence at the neuropsychological but also neuroimaging and biomarker levels.

The experimental designs included cross-sectional analyses to describe the executive performance of the studied population at a given time. In addition, a few longitudinal follow-up and prospective analyses were conducted. The descriptive studies corroborated the existence of a direct relationship between subjective cognitive complaints and the impairment of several executive processes, such as attention, working memory, initiative, cognitive flexibility, inhibition, planning, monitoring, verbal fluency, decision making and goal-directed behaviors [36,39,42,45,46,47,48]. These functions were directly associated with self-reported complaints from participants but also their informants [34,41,43,44]. On the other hand, the characterization of executive performance throughout the evolution of cognitive deterioration, based on cognitive complaints before or after clinical diagnosis, pointed to follow-up studies as essential not only to diagnose and monitor cognitive impairment but, more importantly, to determine a possible predictive relationship with subjective cognitive complaints. Thus, the longitudinal studies [33,35,37,38,40,44] indicated that, in a variable percentage, people presenting subjective cognitive complaints showed a significant cognitive decline in executive processes during the progressive neuropsychological follow-ups, evolving to a possible picture of mild cognitive impairment (MCI) or dementia. In addition, a 15-year prospective study on people with Alzheimer’s disease showed that lower cognitive performance at the executive level started to become more pronounced 2–3 years prior to receiving a clinical diagnosis [35].

The application of standardized neuropsychological tests was the common tool in all the studies, with MMSE as a general screening tool to assess cognitive performance being used in seven of them. However, the review shows that the administration of classical screening tests or questionnaires to study cognitive impairment may not be sufficient for a detailed understanding of executive performance since they are not sensitive enough. Thus, inserting specific neuropsychological tools for executive functions is essential in any clinical analysis. Among them, the most used tests assessed verbal and semantic fluency, cognitive processes such as working memory (digits—WAIS), attentional control (digits, symbol-substitution test and TMT A–B), initiation/perseveration (Korean rating scale K-DRS), BRIEF-A and inhibitory control (Stroop test). On the other hand, not all but some of the studies complemented the neuropsychological assessment with biological markers in the CSF or the use of neuroimaging [37,38,40,43,45,48]. Despite the high cost of neuroimaging studies and their limitations in terms of specificity and power resolution in the initial clinical analysis, which limits their applicability in the population with subjective cognitive complaints, the studies related executive performance to the presence of biomarkers in the CSF and to reductions detected by regional volumetric analyses. Interestingly, a direct relationship between CSF biomarkers present in the preclinical stages of dementia and the impairment of executive processes was found. Thus, higher p-tau values and β-amyloid deposition were associated with subjective cognitive complaints and lower performance in executive functions such as inhibition [37,38,45]. Similarly, neuroimaging studies revealed a direct relationship between subjective cognitive complaints and atrophy of orbital prefrontal regions, reduced functional connectivity in retrosplenial–precuneus regions, reduced temporal cortical thickness (bilateral hippocampi) and left frontal regions, associated with decreased executive performance [38,40,43,45].

## 4. Discussion

The first observation of this systematic review is that, despite the scarcity of literature focusing on the early study of cognitive decline in executive processes, there is a recent growing interest in the prodromal stages of this cognitive domain and the potential predictive value of subjective complaints in this respect. The data corroborate the relationship among subjective cognitive impairment and executive functioning, the relevance of not underestimating the patients’ and/or informants’ complaint reports. However, the data can be considered inconclusive, a fact that may be due to the rather small number of SCI patients used in most of the studies, as well as the heterogeneity of the neuropsychological tools used and multiple variables involved. Consistency regarding the screening tools is needed. Complementing neuropsychological screening with biomarkers and neuroimaging was presented as critical to confirm the early changes in neurological substrates potentially underlying the subjective complaints. Still, despite the complexity of the studies with biomarkers, some studies used almost only MMSE, a test with global acceptance but without the necessary specificity and sensitivity to assess SCI or even MCI patients.

The need for further research and guidelines is enhanced in the current aging population [1] and the global health situation, where the number of middle-aged people with cognitive complaints has dramatically increased [24,25]. The most recent work, a systematic review on the relationship between subjective cognitive complaints and informants’ reports, by Wasef et al. [49] in the context of anesthesiology, provides a general description of the number of cognitive domains affected, with executive functions figuring among them.

Studies assessing executive characteristics in patients with a defined diagnosis, analyzing their impact on a possible prognosis, were more common. For instance, people with amnestic mild cognitive impairment (aMCI) exhibiting frontal executive dysfunction at baseline had overall cortical thinning, including frontal areas, and a higher risk of dementia conversion than those exhibiting visuospatial or language dysfunction [50]. In that clinical scenario, confirming this poor prognosis was essential to determine the higher priority of this subgroup for intervention therapy among aMCI patients. Similarly, proper consideration of the reliability and possible predictive value of subjective cognitive complaints and a better understanding of the early stages of cognitive decline in executive functions would help to define timely interventions in a population that may be at risk. In this sense, comprehensive educational group interventions in community-dwelling older women reporting normal age-related cognitive complaints in the absence of actual cognitive decline were shown to be effective [51]. Complainers receiving psycho-education about cognitive aging and contextual factors, including health, lifestyle, beliefs and negative age stereotypes, reported significatively fewer negative emotional reactions towards cognitive functioning, considered by the authors as a prerequisite for improved subjective cognitive functioning and wellbeing.

Studies analyzing the need to include tools (self-reports) that assess more executive processes such as working memory rather than declarative memory processes also advocated for the relevance of multiple-cognitive-domain screening compared to classical unidimensional cognitive assessments [52]. Thus, one of the limitations in the analyzed studies was the generalization in neurocognitive assessments. Despite the use of more-or-less-specific tests for executive functioning, the study of memory as a unitary process and predictor of dementia continued to be prioritized. However, authors such as Giovanello and Verfaille [53] considered the need to study memory as a multimodal process related to other cognitive functions, making it possible to analyze the relationship of this process with cognitive impairment in-depth. In this sense, the study of specific memory modalities would be associated with executive processes and the activation of frontal regions, which could be associated with preclinical stages of dementia. Types of memory such as prospective memory and metamemory associated with self-awareness of illness are processes that have been investigated in recent years concerning cognitive complaints and executive functioning, as they share a common anato-functional niches, such as the prefrontal cortex [54,55,56,57].

In the same vein, self-reports and reports from external informants can work as sentinels providing valuable information about executive functioning at preclinical stages. Fogarty (2017) [41] stated that people with mild AD had more significant concerns regarding their executive functions associated with their daily lives, an opinion which was corroborated, to a lesser extent, by their informants. However, other studies indicated that only self-reported cognitive complaints predicted future cognitive changes and were associated with executive functioning, unlike informants’ reports being associated with other cognitive processes [58,59].

Highly heterogeneous variables influence subjective cognitive complaints, contributing to underestimating the reliability of this psychological construct. Emotional factors and affective disorders, including depression and anxiety, negatively influence memory but also executive performance. To monitor their contribution, some of the revised studies included neuropsychiatric symptoms in clinical assessments [60,61]. In addition, personality factors can directly influence the self-perception of everyday failures, which could be associated with executive problems [62]. Still, none of the revised works on subjective cognitive complaints and executive functions considered nor assessed the contribution of age stereotypes. In fact, only a few research reports in the literature have specifically studied subjective cognitive complaints and age stereotypes [12,63]. Lubitz et al. [63] recently developed and assessed the psychometric properties of a new questionnaire on subjective cognitive complaints in multiple cognitive domains, its association with psychological variables and the distinction of complainer types. Their study confirmed the strong influence of depressiveness on the overall level of subjective cognitive complaints. Most importantly, it detected that people with complaints about executive functions exhibited the highest levels of affective disorders, were younger and had less social integration.

Depressive symptoms and memory impairments are associated with heightened stress hormone levels during aging; conversely, long-term exposure to high endogenous levels of glucocorticoids is associated with both memory impairment and smaller hippocampal volume. In this context, Sindi et al. [12] studied the role of internalized negative aging perceptions and found them to be associated with increased depressive symptoms and subjective—but not objective—memory complaints. Interestingly, negative age stereotypes did not predict increased cortisol levels, suggesting that the mechanism underlying the association between stereotypes and cognitive impairments may be independent of age-related dysregulations of cortisol secretion [12]. However, although depression and stress contribute to cognitive performance, almost none of the studies used as an obligatory criterion the absence of psychiatric comorbidity.

The studies analyzed in the present review did not provide conclusive data about the relationship among sociodemographic variables, such as gender and level of education, that may influence the individual’s subjective perceptions and executive impairment. Nevertheless, gender medicine considers essential to analyze the effects of sex and gender [64,65] and a recent research study found significant differences in executive performance between men and women with subjective cognitive complaints, with men performing worse than women [66]. On the other hand, in the study, there was minimal specificity of participants’ level of education and its relationship as a possible predictor of dementia. In this sense, we recently confirmed the relevance of considering the interactions between sex/gender and education factors, since a lower level of education in women can be a critical risk factor for dementia in vulnerable subjects [67].

## 5. Conclusions

In conclusion, the study of subjective cognitive complaints and their relation to executive functioning draws attention to several dimensions of clinical interest; therefore, it can be foreseen to be a promising emerging field. However, the specific search for information in this regard will find limitations as long as the term “subjective cognitive complaints” remains ambiguous and does not have a precise definition. Psychiatric comorbidity should be considered as a confounding factor, thus an exclusion criterion. Studies including a rather small number of SCI patients, no consistency regarding tools and the general use of MMSE are other limitations of the current literature.

The studies corroborated the relationship between subjective cognitive complaints and some executive processes, which is noteworthy since many people with subjective executive complaints progress to dementia. The relational studies confirmed that impaired executive performance was associated with CSF biomarkers and reduced cortical volume in specific brain regions. However, it is necessary to focus on unifying the screening tools based on processes rather than isolated functions when studying the significant heterogeneity involved in this preclinical construct. Applying an adequate neuropsychological assessment in parallel to the clinical study and a detailed analysis of sociodemographic variables would allow a better definition of the neurocognitive profile of adults who report executive complaints in their daily lives to be obtained. Otherwise, there is a risk of underestimating the reliability and predictive value of self-reporting, which may hamper the chances of providing an adequate diagnosis and early and timely intervention.

## Figures and Tables

**Table 1:** Summary of the studies analyzing the relationship between subjective cognitive complaints and executive functions.

Authors	Sample	Methodology	Results
Rapp and Reischies (2005) [33]	187 participants (83 W and 84 M): 15 cases who developed AD (7 W and 8 M) Mean age: 86.17 years Mean years of education: 11.43 172 cases who did not develop AD (76 W and 76 M) Mean age: 79.08 years Mean years of education: 11.30 Country: Germany	Longitudinal study (4 years) NEUROPSYCHOLOGY MMSE, TMT part B, Digit Symbol Substitution Test, Digit Letter test, Identical Pictures	In total, 15 participants developed Alzheimer’s disease at 4 years (scoring low on all follow-up assessments).
Rabin et al. (2006) [34]	87 participants (54 W and 33 M): 30 HC subjects (21 W and 9 M) Mean age: 72 years Mean years of education: 17 28 SCC subjects (20 W and 8 M) Mean age: 74 years Mean years of education: 16 29 MCI subjects (13 W and 16 M) Mean age: 74 Mean years of education: 16 Country: United States	NEUROPSYCHOLOGY MMSE, CVLT-II, DRS-2, WCST, D-KEFS, WMS-III, BRIEF-A	The participants with MCI and SCCs reported significant difficulties with selective aspects of executive functioning (working memory). In addition, it was more likely that their informants reported the same difficulties.
Grober et al. (2008) [35]	92 subjects with incident AD assessed for 15 years prior to diagnosis (48 W and 44 M) Mean age: 79.8 years Mean years of education: 16.5 Country: United States, Netherlands	NEUROPSYCHOLOGY Memory (Free Recall on the Free and Cued Selective Reminding Test), executive functions (categorical fluency, letter and path fluency), and verbal intelligence (AMNART)	The decline in the cognitive performance in episodic memory tests accelerated 7 years prior to diagnosis; this was 2–3 years for executive-function performance (associated with pathological signs in the frontal circuits), while verbal-intelligence performance declined in the vicinity of diagnosis.
Saunders and Summers (2010) [36]	131 subjects (68 W and 63 M): 25 HC subjects Mean age: 69 years Mean years of education: 13.5 32 subjective MCI Mean age: 71 years Mean years of education: 13 60 amnestic MCI Mean age: 71 years Mean years of education: 13.1 14 mild AD Mean age: 76 years Mean years of education: 12 Country: Australia	NEUROPSYCHOLOGY Wechsler Test of Adult Reading (WTAR); estimated full-scale intelligence quotient (FSIQ); Boston Naming Test (BNT); Rey Auditory Verbal Learning Test (RAVLT); Paired AssociateLearning (PAL).	Both the amnestic-MCI and subjective-MCI groups displayed impaired attentional processing and working-memory capacity.
van Harten et al. (2013) [37]	132 participants with SCCs (56 W and 76 M) Mean age: 61.4 years Mean years of education: 6 Country: Netherlands	NEUROPSYCHOLOGY Neuropsychological assessments with 1–2 years follow-up (MMSE) CSF BIOMARKERS CSF biomarker data on β-amyloid, total tau, hyperphosphorylated tau-181	Patients meeting preclinical AD criteria showed deterioration of memory, executive functions and global cognition over time, associated with evidence of CSF. MMSE total scores, delayed recall and initiation/perseveration subscales were significantly lower than the control group.
Toledo et al. (2015) [38]	522 subjects (253 W and 269 M): 307 CN subjects (138 W and 169 M) Mean age: 73.9 years 71 subjects with SCCs (25 W and 46 M) Mean age: 71.6 years 51 subjects with executive SCI (21 W and 30 M) Mean age: 77.3 years 66 subjects with memory SCI (49 W and 17 M) Mean age: 75 years 27 subjects with multi-domain SCI (20 W and 7 M) Mean age: 78 years Country: United States	CSF BIOMARKERS Analysis of CSF biomarker data NEUROIMAGING Hippocampal volume measurements and MRI-SPARE-AD values Hypometabolic convergence index Posterior cingulate metabolic rate for glucose	Conversion from executive SCI to MCI/dementia of 50% at 7 years. Participants with SCCs showed atrophy of orbital prefrontal regions and higher p-tau and RM-SPARE-EA values (indicative of pathological changes).
Seo et al. (2016) [39]	265 participants (178 W and 79 M): 188 CN subjects (120 W and 60 M) Mean age: 71.94 years Mean years of education: 9.69 77 subjects with pre-DCL (58 W and 19 M) Mean age: 72.64 years Mean years of education: 9.64 Country: Korea	NEUROPSYCHOLOGY MMSE; categorical and phonemic fluency tests, Stroop test, path test and TMT A–B	Significantly lower pre-DCL-group scores in visual memory and executive functions, with poor performance in inhibition and goal-directed behavior.
Verfaillie et al. (2016) [40]	238 participants with SCI (109 W and 129 M) Mean age: 62 years Country: Netherlands	NEUROPSYCHOLOGY Neuropsychological assessment (MMSE) NEUROIMAGING Measurement of cortical thickness by MRI	A total of 16% of participants showed progression to MCI and AD at the 2–3 year follow-up. Reduced fronto-temporo-parietal cortical thickness associated with reduced memory performance. Reduced temporal cortical thickness was associated with decreased executive performance.
Fogarty et al. (2017) [41]	55 participants (35 W and 20 M): 23 subjects with mild AD (9 W and 14 M) Mean age: 73.95 years Mean years of education: 15.56 32 adult control subjects (26 W and 6 M) Mean age: 69.84 years Mean years of education: 13.96 Country: England	NEUROPSYCHOLOGY Application of the BRIEF-A to participants and informants (spouse/child)	Participants with mild AD and their informants reported greater difficulties in most of the clinical scales assessed.
Bae et al. (2017) [42]	1442 participants 1088 HC subjects 354 with SCCs Country: South Korea	NEUROPSYCHOLOGY MMSE, initiation/perseveration (IP), Subscale of the Korean version of Mattis Dementia Rating Scale (K-DRS)	Participants with SCCs performed worse on all cognitive tests that evaluated memory and executive functions.
Viviano et al. (2018) [43]	83 participants (51 W and 32 M): 35 adults with SCI (22 W and 13 M) Mean age: 68.5 years 48 adults without SCI (29 W and 19 M) Mean age: 67.08 years Country: United States, Netherlands	NEUROPSYCHOLOGY MMSE and Weschler Memory Scale, Geriatric Depression Scale, Beck Depression Inventory II and Big Five Inventory NEUROIMAGING Assessment of functional connectivity of brain regions using MRI and diffusion of regions	Patients and informants were more likely to report executive problems in working memory, planning/organising and monitoring. Subjective cognitive impairment was associated with lower functional connectivity in retrosplenial–precuneus regions and memory-system regions (poorer performance in visual working memory).
Valech et al. (2018) [44]	68 normal subjects (46 W and 22 M): 52 HC (33 W and 19 M) Mean age: 63.87 years Mean years of education: 11.96 16 pre-AD (13 W and 3 M) Mean age: 66.5 years Mean years of education: 9.56 Country: Spain	Neuropsychological follow-up for 1 year NEUROPSYCHOLOGY Subjective Cognitive Complaints Questionnaire (SCI-Q) MMSE, Memory Alteration Test, Boston Naming Test, comprehension of commands (BDAE), incomplete letters and number location (VOSP), TMT-A, phonetic fluencies (FAS), Free and Cued Selective Reminding Test (FHCRT-IR), semantic fluency test (animals), Stroop test Hospital Anxiety and Depression Scale	Pre-AD subjects showed significantly higher scores with respect to language, attention and executive decline, confirmed by their informants. Significantly decreased cognitive performance in pre-AD on tests of inhibition and semantic fluency.
Pérez et al. (2020) [45]	195 participants with SCD (121 W and 74 M) Mean age: 65.71 years Mean years of education: 14.94 Country: Spain	NEUROPSYCHOLOGY TMT A-B, rule shift card subtest (BADS), automatic inhibition subtest (AI-SKT), digits (WAIS-III), letter, semantic and verbal fluency tests NEUROIMAGING Functional neuroimaging studies (positron emission tomography with 18F-Florbetaben and MRI) Analysis of gray matter volume	Significant association between β-amyloid deposition and low executive performance in automatic inhibition (AI-SKT). Low executive performance was associated with lower volume in bilateral hippocampal and left frontal regions.
Kim et al. (2020) [46]	1442 participants (886 W and 556 M) (age: ≥65 years): 1088 HC subjects (642 W and 446 M) Mean years of education: 5.66 354 SCC subjects (244 W and 110 M) Mean years of education: 3.33 Country: Korea	NEUROPSYCHOLOGY Mini-Mental State Examination, Korean version (MMSE-KC)—use of the recording and retrieval subscales for memory assessment and the initiation/perseveration subscales (Korean rating scale K-DRS) to assess executive functions	Significant relationship between depression and SCCs. Lower performance in global cognition, memory and executive functions (verbal fluency) in the study group.
Esmaeili et al. (2021) [47]	62 subjects: 17 SCC subjects 30 amnestic-MCI subjects 15 HC subjects Country: Iran	NEUROPSYCHOLOGY Attention Network Test (ANT)	The older SCC subjects faced problems in maintaining alertness to external stimuli (attention-processing problems).
Garrido et al. (2021) [48]	136 subjects (67 W and 59 M): 28 young adults with SCCs (17 W and 11 M) Mean age: 21 years 37 young adults without SCCs (16 W and 11 M) Mean age: 23 years 32 older adults with SCCs (18 W and 14 M) Mean age: 63 years 39 older adults without SCCs (16 W and 23 M) Mean age: 65 years Country: Spain	NEUROPSYCHOLOGY Iowa gambling task (IGT) NEUROLOGY Electroencephalogram (EEG) Feedback-related negativity (FRN)	The older adults with SCCs presented deficits in the decision-making process. These data were also observed in the neuronal mechanisms studied.

Abbreviations: W, women; M, men; SCCs, subjective cognitive complaints; SCD, subjective cognitive decline; AD, Alzheimer’s disease; HC, healthy control; CSF, cerebrospinal fluid; CN, cognitively normal; SCI, subtle cognitive impairment; MRI-SPARE-AD, spatial pattern abnormalities for early Alzheimer’s recognition; MRI, magnetic resonance imaging; MCI, mild cognitive impairment.

## Data Availability

Not applicable.
